# Immediate Antiretroviral Therapy: The Need for a Health Equity Approach

**DOI:** 10.3390/ijerph17197345

**Published:** 2020-10-08

**Authors:** Ofole Mgbako, Magdalena E. Sobieszczyk, Susan Olender, Peter Gordon, Jason Zucker, Susan Tross, Delivette Castor, Robert H. Remien

**Affiliations:** 1 Division of Infectious Disease, Department of Internal Medicine, Columbia University Irving Medical Center, New York, NY 10032, USA; mes52@cumc.columbia.edu (M.E.S.); so2045@cumc.columbia.edu (S.O.); pgg2@cumc.columbia.edu (P.G.); jz2700@cumc.columbia.edu (J.Z.); dc2022@cumc.columbia.edu (D.C.); 2 HIV Center for Clinical and Behavioral Studies, NY State Psychiatric Institute and Columbia University, New York, NY 10032, USA; st130@cumc.columbia.edu (S.T.); rhr1@cumc.columbia.edu (R.H.R.)

**Keywords:** HIV, antiretroviral therapy, rapid, health equity

## Abstract

Immediate antiretroviral therapy (iART), defined as same-day initiation of ART or as soon as possible after diagnosis, has recently been recommended by global and national clinical care guidelines for patients newly diagnosed with human immunodeficiency virus (HIV). Based on San Francisco’s Rapid ART Program Initiative for HIV Diagnoses (RAPID) model, most iART programs in the US condense ART initiation, insurance acquisition, housing assessment, and mental health and substance use evaluation into an initial visit. However, the RAPID model does not explicitly address structural racism and homophobia, HIV-related stigma, medical mistrust, and other important factors at the time of diagnosis experienced more poignantly by African American, Latinx, men who have sex with men (MSM), and transgender patient populations. These factors negatively impact initial and subsequent HIV care engagement and exacerbate significant health disparities along the HIV care continuum. While iART has improved time to viral suppression and linkage to care rates, its association with retention in care and viral suppression, particularly in vulnerable populations, remains controversial. Considering that in the US the HIV epidemic is sharply defined by healthcare disparities, we argue that incorporating an explicit health equity approach into the RAPID model is vital to ensure those who disproportionately bear the burden of HIV are not left behind.

## 1. Introduction

Gaps in the HIV care continuum, particularly between HIV diagnosis, linkage to HIV care, and initiation of antiretroviral therapy (ART), present a persistent global public health challenge [1]. Multiple medical advancements in HIV care aim to mitigate drop-off along the care continuum. Early ART, regardless of CD4 count, has been shown to decrease morbidity and mortality among patients living with HIV [2,3]. Universal test and treat strategies have been shown to reduce the risk of sexual transmission of HIV from virologically suppressed individuals to their sexual partners, also known as treatment as prevention [4,5,6]. At the same time, the advent of new potent antiretrovirals, including integrase inhibitors, have resulted in regimens that are easier to tolerate, with fewer drug–drug interactions, higher barriers to resistance, and more single-dose regimen options [7]. These advances led to the exploration of rapid or immediate ART (iART)—defined as same-day, or as soon as possible after diagnosis, ART without waiting for initial bloodwork or viral resistance testing—as a potential approach to close the gap between HIV diagnosis, linkage to care, and ART initiation. 

Immediate ART emphasizes the primacy of treatment. The conceptual framework of iART moves away from the “patient readiness” model, in which initiation of ART after diagnosis often can take weeks or even months as providers assess factors such as a patient’s psychological state, coping abilities, support system, and knowledge about HIV [8]. While iART has led to generally positive short-term outcomes, including time to viral load suppression and time to linkage to care, the impact of iART on long-term outcomes, including ART adherence and retention in care, is still under exploration [9,10,11]. Indeed, some studies have shown negative impacts on retention in care among patients who receive iART [12,13,14]. 

Emerging implementation approaches of iART in the US, like San Francisco’s Rapid ART Program Initiative for HIV Diagnoses (RAPID) model, have successfully condensed insurance acquisition, housing assessment, and mental health and substance use evaluation into an initial visit [15]. However, even the RAPID program, which serves a highly vulnerable patient population, can not explicitly address all psychosocial factors, such as structural racism, homophobia, HIV-related stigma, trauma, medical mistrust, and other important considerations present for patients at time of diagnosis. The HIV epidemic in the US is driven by significant disparities among racial/ethnic, gender, and sexual minorities [16]. There is a lack of evidence regarding how these patients perceive and experience iART, as well as iART’s impact on these aforementioned psychosocial factors. A more thorough examination of iART’s impact, particularly for the most vulnerable populations, is critical to meet the goals of ending the epidemic and to ensure that in addition to the universal, immediate offer of treatment, there is an appreciation of and targeted interventions for specific patient concerns. This commentary argues that a health equity approach that recognizes and intervenes upon the psychosocial forces impacting the most vulnerable populations living with HIV is vital for successful iART implementation and to avoid exacerbating disparities along the HIV care continuum.

## 2. The Global Move towards Immediate Antiretroviral Treatment

Multiple international studies comparing same-day ART initiation to standard of care have found that same-day initiation was associated with improved viral suppression and retention in HIV care. The CASCADE trial from Lesotho found that a same-day home-based ART program compared to usual care resulted in significant increases in linkage to care at 3 months and viral suppression at 12 months [11]. A retrospective study among patients with acute HIV infection in London showed that of those who started ART at their first medical appointment, none had discontinued ART and nearly all achieved viral suppression at 24 weeks [10]. In a study conducted in Haiti, Koenig et al. compared clinical outcomes in patients starting same-day ART to a control group initiating ART 3 weeks after diagnosis, with the composite primary outcome being retention in care with a suppressed viral load at 12 months [9]. They found a statistically significant difference in the composite outcome in favor of those initiating same-day ART [9]. 

In 2017, based on the strength of evidence from these studies, the World Health Organization (WHO) management guidelines endorsed iART, defined as starting ART within 1 week of HIV diagnosis [17]. Following this, clinics throughout sub-Saharan Africa, and other regions of the world with high HIV burden and low patient-to-provider ratios, began to figure out ways to speed up ART initiation through innovative programs. For example, the Streamlined ART START-ART Strategy in Uganda called for opinion-leader-led trainings of healthcare providers on the benefits of immediate initiation, coaching of doctors and nurses on how to assess individual “readiness,” and the expansion of point-of-care CD4 count assays and other diagnostics to rural clinics [18]. As programs in low- and middle-income countries focused on provider comfort with and education about iART and effectiveness of medication delivery systems, iART became a more accepted approach in many parts of the world [18]. 

While many international studies have shown promising results along the HIV care continuum for iART, others have had uneven results. A recent meta-analysis, for example, highlighted an increase in loss to follow-up in some cases [19]. In South Africa and Kenya, Rosen et al. conducted a study in which they randomized newly diagnosed patients with HIV to an intervention called SLATE (Simplified Algorithm for Treatment Eligibility), which involved conducting a patient history, a physical exam, and a readiness assessment for same-day initiation versus usual care [12]. The SLATE intervention increased ART uptake at 1 month, however, there was no effect on retention in care at 8 months in either South Africa or Kenya [12]. A more recent observational study from France showed that newly diagnosed patients starting ART less than 9 days after HIV diagnosis had higher loss to follow-up at 1 year than those who had started ART greater than 90 days after HIV diagnosis [13]. The authors pointed to a need to understand the “social and life context” of the population with increased loss to follow-up, surmising that issues of migration and stigmatization among immigrants from sub-Saharan Africa in France may have contributed to poor outcomes [13]. Yet another recent prospective cohort study from South Africa showed that patients initiated on same-day ART had an increased risk of loss to follow-up compared to those initiating >1 day [14]. These data point to lack of current consensus in the literature regarding iART’s impact on long-term outcomes. There is debate whether even measuring the impact of iART on long-term outcomes is warranted, given that the same barriers to viral suppression and retention in care likely exist for patients regardless of iART. However, a patient’s initial experience after diagnosis, the immediate offer of treatment, and the introduction into the HIV clinical care setting likely does have some impact on long-term engagement [20]. This points to the need for a mindful approach in the implementation and rollout of iART, despite the consensus among international clinical care guidelines. 

Qualitative studies have provided a deeper understanding of concerns about iART for longer-term outcomes, particularly among vulnerable groups. While many qualitative studies in the global context have shown that patients find iART acceptable, some studies signal concerns. Helova et al. used in-depth interviews and focus groups to evaluate an Option B+ program (i.e., providing ART continuously starting in the pre-natal period) in Uganda and found that pregnant women living with HIV and their male partners more often than not accepted an iART approach, but at times felt uncomfortable with same-day initiation due to lack of time for diagnosis acceptance and discussion with family [21]. Similarly, Katirayi et al., in evaluating an Option B+ program in Malawi, showed pregnant women with HIV preferred to discuss their circumstances with their husbands prior to same-day initiation, and often wanted to check a CD4 count and repeat an HIV test at another facility to confirm their HIV status [22]. Maek-a-nantawat et al. assessed attitudes among men who have sex with men (MSM) in Thailand about iART pre- and post-HIV diagnosis and found some concerns about medication cost and side effects, as well as the concern that others would discover their diagnosis despite their high interest in iART [23]. These concerns do not mean there should be a return to the “patient readiness” model of the past, rather they call for a patient-centered approach that balances iART and its proven benefits (e.g., shorter time to viral load suppression) with an effort to empower patients to face the significant structural barriers to positive HIV care outcomes. 

## 3. The RAPID Model, Expansion of iART, and Health Disparities in the US Epidemic

There has been an accelerated paradigm shift towards iART both globally and in the US. While a recent global meta-analysis of iART studies emphasized that the strongest evidence supporting favorable HIV care outcomes comes from low- and middle-income countries, there have been no randomized controlled trials of iART programs in the US [24]. A clinic-based cohort study of San Francisco General Hospital’s Ward 86 RAPID model, the first noted iART program in the US, showed improved time to viral suppression and time to linkage to care with iART versus delayed ART [15]. About 95% of patients who underwent RAPID initiated ART the same day as HIV diagnosis and reached viral suppression at 1.8 months compared to non-RAPID patients who reached viral suppression at 4.3 months [15]. 

Observational studies of the San Francisco iART experience identified outcome disparities between subgroups. Follow-up of the RAPID cohort did show that 95% maintained viral suppression at 1 year [25]. However, as this approach was adopted by the entire city of San Francisco, a recent evaluation of citywide RAPID outcomes demonstrated that some groups, including Latinx patients, were more likely to initiate iART at first visit, while transgender women were least likely to initiate iART at first visit [26]. There were also emerging disparities in time to first viral suppression among people who inject drugs and African Americans [26]. The signal of emerging disparities highlighted by this study calls for an evaluation of what key elements need to be added to the RAPID model to better serve the most vulnerable populations with HIV.

Following the RAPID study, observational studies across the country have shown similar improvement in initial outcomes after iART. In a retrospective study, Hoenigl et al. showed that among newly diagnosed patients with HIV in San Diego, CA, those who initiated same-day ART were more likely to be virologically suppressed at 12 weeks [27]. Colasanti et al. also showed a reduction in median time to viral suppression for iART patients at a clinic in Atlanta, GA [28]. Rodriguez et al. showed nearly all iART patients remained virally suppressed at 1 year in a Miami, FL, clinic [29]. Also, Halperin et al. reported high rates of retention in care and viral suppression from a rapid-start program in New Orleans, LA, in both newly diagnosed patients and patients who were ART naïve and not immediately linked to care [30]. It is important that these observational results have been replicated in different regions of the country, with racially and socioeconomically diverse patient populations. However, with iART becoming standard of care in many US jurisdictions, it is vital to understand how iART impacts the most vulnerable populations along the entire HIV care continuum, particularly given concerns about iART’s impact on long-term outcomes in the global context and potential emerging disparities in iART uptake among the RAPID cohort. 

The HIV epidemic in the US is currently marked by significant disparities among racial/ethnic, gender, and sexual minorities. Out of the over 38,000 new HIV infections in 2017, 70% were among gay and bisexual men [31]. In 2018, African Americans made up 42% of new HIV infections while only comprising 13% of the population, and HIV infections increased among Latinos in all 50 states from 2010 to 2016 [31,32]. People living with HIV who lie at the intersection of these identities see the most glaring disparities. Among all MSM, Black MSM account for 37% of new HIV diagnoses, with growing concentration in the South, and fare worse at each step along the HIV care continuum (e.g., linkage, retention, and viral suppression) compared to their White counterparts [16]. Half of Black MSM are projected to acquire HIV in their lifetime [33]. Nearly half of all Black transgender women and a quarter of all Latina transgender women are estimated to be living with HIV [34]. Given that iART may quickly medicalize the post-diagnosis experience for some patients, potentially in advance of emotional processing, it is critical to understand iART’s impact in these communities to avoid the potential of emerging HIV-related health disparities. Consider, for example, that many well-intentioned major advancements in HIV prevention, care, and treatment (e.g., pre-exposure prophylaxis (PrEP), widespread testing, advances in antiretroviral treatment) have not improved racial disparities in HIV care outcomes. While improving time to viral suppression, iART has the potential to further racial disparities if not implemented with a deep understanding of how Black people living with HIV understand and perceive the iART intervention.

Major national clinical care guidelines from the US Department of Health and Human Services officially have endorsed an iART approach because of its clear benefits in achieving viral suppression more quickly [35]. The current debate focuses on the optimal timing of ART—whether same-day, or “as soon as possible” is sufficient; however, the discussion should not ignore how iART may worsen disparities for vulnerable populations. We agree that a universal approach defined by immediate initiation is more optimal than a universal approach defined by the “readiness” models of the past. However, as we incorporate universal iART into clinical care delivery models, the specific concerns of the most marginalized groups (whether it be racism, HIV-related stigma, medical mistrust, health literacy, or other factors) should also be assessed and intervened upon. The RAPID model, upon which most US iART programs are based, addresses certain important structural and behavioral factors that drive disparities. However, it does not, to our knowledge, address some known psychosocial factors, including HIV-related stigma and medical mistrust at diagnosis, that disproportionately affect the groups disproportionately impacted by HIV. A health equity approach focused on allaying disparities will be integral to successful ongoing iART implementation for all patient populations in varied geographic contexts.

## 4. A Health Equity Perspective for iART: Stigma and Mistrust among Racial, Gender, and Sexual Minorities

Health equity “implies that resources are distributed and processes are designed in ways most likely to move toward equalizing the health outcomes of disadvantaged social groups with the outcomes of their more advantaged counterparts” (p. 257) [36]. In the context of iART, a health equity approach first requires a full and deep understanding of all of the factors that drive disparities at each juncture of the HIV care continuum. It then necessitates the appropriate distribution of resources, as well as targeted policies and interventions for the most disadvantaged groups of people living with HIV.

Little is known about the psychosocial costs and benefits of iART after diagnosis among racial/ethnic, gender, and sexual minorities. There is a substantial risk for exacerbating existing disparities if iART’s impact on the most vulnerable communities is not fully taken into account. HIV-related stigma, discrimination, and medical mistrust are some of the most significant barriers to care along the HIV care continuum for these populations. One can imagine that factors such as medication beliefs, health literacy, and other issues may also arise at time of diagnosis and further complicate an iART approach. In order to avoid further HIV-related disparities among racial, gender and sexual minorities, one must address these psychosocial factors in patients’ lives in the context of iART. 

Following an HIV diagnosis, HIV-related stigma has a deleterious impact on HIV care engagement for minority patients. Turan et al. adapted the minority stress model to create a framework elucidating the multiple dimensions of HIV-related stigma at the individual level, including enacted (e.g., experienced discrimination), internalized (e.g., shame), anticipated (e.g., expectation of discrimination), and perceived (e.g., perception of stigmatized attitudes in the community) HIV stigma [37]. The framework also includes both structural stigma and intersectional stigma, which acknowledges that macro-level factors are inextricably linked to individual-level stigma [37]. Studies have explored the effects of different types of individual HIV-related stigma on HIV-related outcomes. One study among young Black MSM showed internalized HIV stigma was associated with lower rates of viral suppression [38]. Another study among Black and Latino heterosexual adults showed that anticipated HIV stigma led to lower HIV care engagement [39]. These different forms of individual level HIV-related stigma disrupt coping processes, diagnosis disclosure, and establishment of social support networks, undermining medication adherence and engagement with HIV care services [40,41]. Immediate initiation may alleviate stigma and concern about one’s HIV status and empower patients through early integration of a new diagnosis. Alternatively, it may worsen ongoing stigma processes. Either way, mitigating stigma in its many dimensions must be considered to ensure iART’s successful implementation.

Mistrust of the healthcare system is another barrier to engagement in HIV care for minority communities, especially given the complicated history of the US healthcare system with marginalized populations (e.g., medical experimentation on African Americans) [42]. A study of African Americans’ perceptions of HIV care showed that distrust of providers led to lack of care engagement [43]. Indeed, studies have explored the association between medication necessity beliefs and medical mistrust, and how together they can negatively impact ART adherence [44]. Another recent study among Black MSM found that nearly half reported existing mistrust of the medical establishment [45]. Suspicion of healthcare provider motive and HIV treatment decisions may persist in the context of iART, given the emphasis on medication, and it is imperative to understand whether iART allays concerns regarding mistrust in the healthcare system or exacerbates existing beliefs around experimentation and ulterior motive.

While iART could potentially exacerbate stigma and mistrust for some patients, it equally has the potential to begin to address HIV-related health disparities for racial, gender, and sexual minorities. In 2010, Grimes et al. provided a literature review showing that a “readiness” model lacked utility in improving HIV care outcomes [46]. As the efficacy of current ART made emerging viral resistance less of a concern, and the benefits of early ART have become clear, iART provides a strong foundation upon which to build an intervention at HIV diagnosis that prioritizes patient-centeredness and health equity.

It is also important to note that the iART approach has extended not only to newly diagnosed patients living with HIV, but also patients who seek to re-engage in care after a long period of time off treatment. This extremely vulnerable population requires a more sensitive approach that considers the forces of stigma and mistrust. Arguably, they may well be an even more vulnerable, and more hidden, population than the newly diagnosed—without highly visible, tailored programs for them. A recent qualitative study by Freeman et al. showed that Black and Latino patients living with HIV who were out of care viewed HIV care decisions through the lens of institutional discrimination, which led to feeling pressured to take ART, excluded from shared-decision making, and ignored in terms of other life concerns [47]. Incorporating a health equity perspective focused on eliminating disparities into the iART intervention must include the perspectives of patients who continually fall out of HIV care. An iART approach when these patients return to the clinical setting may serve to push them further away if issues of stigma and mistrust are not explicitly addressed.

## 5. Future Directions

In a review of iART in 2019, Boyd et al. called for more high-quality studies to be conducted on iART in high-income countries and for an evaluation of its impact on patient empowerment [48]. Indeed, for programs that have not yet adopted iART, a randomized control trial evaluating clinical outcomes would help bolster the evidence base for iART as a proven intervention for the HIV care continuum in the US. In addition, implementation science studies will be vital to glean best practices as organizations move to an iART model in different clinical care delivery settings (e.g., large academic hospital clinics, community-based clinics, federally qualified health centers) with the goal of achieving health equity. Understanding how iART is operationalized (e.g., same-day visits, role of patient navigators) in different clinical contexts and what kinds of interventions have made a difference with at-risk populations will also provide important guidance. There is room for more tailored approaches depending on the context of different cities and states and their unique healthcare infrastructure (e.g., Medicaid expansion) and patient population needs (e.g., undocumented patients).

In addition to stigma and medical mistrust, which are well-documented in the literature, future studies must identify the role other psychosocial factors play with iART. The role of structural racism and homophobia, and other forms of structural discrimination—that is, in the form of cultural norms and institutional policies—and their interplay with iART is an important area of potential inquiry [49]. Health literacy is yet another issue that influences HIV health outcomes and may carry particular resonance in the context of iART and health equity, given the need to immediately understand and agree with a pharmacological intervention (i.e., ART), as well as the complexity of navigating the health system for patients living with HIV [50]. 

It also needs to be taken into account that an HIV diagnosis may be traumatic for vulnerable groups given the history of the HIV/AIDS epidemic, and the disproportionate loss of life in the LGBTQ community and in communities of color [51,52]. Trauma, a stressful life event marked by a sense of horror, helplessness, serious injury, or threat of serious injury or death, can lead to poorer HIV health-related outcomes [52,53]. Thus, it is important to explore the trauma that patients who are newly diagnosed or returning to clinical care need addressed in the context of the iART intervention. Notably, mental health assessment is a significant part of the RAPID model, and ideally symptoms of trauma would be identified during iART and patients would be linked with appropriate mental health services. Yet an honest assessment of these symptoms among racial/ethnic, gender, and sexual minorities is often hindered by medical mistrust or levels of stigma. Thus, optimizing the current model and aspiring towards health equity require not simply a universal approach, but a targeted assessment and screening of factors that affect the most vulnerable.

Shared decision making may be an important area of exploration in the context of iART, given that patients living with HIV from vulnerable populations often expect that they will not have a say in their treatment plan and alternatively feel empowered when they do [48,54]. True shared decision making creates the space and possible framework for the exploration of stigma, alternative medical beliefs, structural racism, medical mistrust, health literacy, and a myriad of person-centric priorities rather than physician-centric priorities. Shared decision making can be a potential tool that allows these concerns to emerge upfront before iART. It may also serve to allay organizational stigma, given that patients may feel stigmatized for not accepting iART or may even be treated differently for not accepting iART. Indeed, a continual evaluation of stigma in health facilities will be an important practice in a health equity model [55].

Lastly, there is growing recognition that qualitative research enriches our understanding of the acceptability, context, barriers, and facilitators of HIV care and treatment interventions [56]. To understand how vulnerable patients cope with a new HIV diagnosis in the context of iART, as well as the promise and perils of iART in a diverse population in terms of race, ethnicity, gender, and sexual orientation, an in-depth exploration of the role of stigma and mistrust is required. This deep understanding gained from qualitative research may help devise effective interventions that lay the foundation for an iART model rooted in health equity. 

In addition, there are potential theoretical frameworks that may help researchers incorporate a health equity lens into research focused on iART. For example, critical race theory may provide a basis for the qualitative exploration of the impact of structural racism on HIV diagnosis and immediate offer of treatment, and allow for targeted interventions in the context of iART [48]. In terms of implementation, there is also potential for the integration of screening tools at diagnosis to know which psychosocial issues to prioritize along with universal iART. Issues of stigma, mistrust, trauma, and low health literacy may be invisible to providers at time of diagnosis, thus an explicit exploration of how these structural issues affect iART for patients at the individual level provides an opportunity for a more targeted approach as we seek to end the HIV epidemic.

## 6. Conclusions

Immediate ART is being scaled up around the country. Yet, if a disparities focus targeting individual- and structural-level inequities among racial, gender, and sexual minorities is not at the forefront of the iART intervention, we face the risk of leaving behind the most at-risk and most vulnerable populations living with HIV. As the US makes strides in decreasing overall HIV incidence, racial, gender, and sexual minorities bear a greater burden of HIV and experience poorer outcomes along the HIV care continuum. A health equity approach to iART is vital to its successful rollout and implementation, ensuring we close the gap along the HIV care continuum for the most marginalized people living with HIV. 

## References

[1] Rosen S., Fox M.P. (2011). Retention in HIV care between testing and treatment in sub-Saharan Africa: A systematic review. PLoS Med..

[2] Lundgren J.D., Babiker A.G., Gordin F., Emery S., Grund B., Sharma S., Avihingsanon A., Cooper D.A., Fätkenheuer G., INSIGHT START Study Group (2015). Initiation of antiretroviral therapy in early asymptomatic HIV infection. N. Engl. J. Med..

[3] The TEMPRANO ANRS 12136 Study Group (2015). A trial of early antiretrovirals and isoniazid preventive therapy in Africa. N. Engl. J. Med..

[4] Cohen M.S., Chen Y.Q., McCauley M. (2011). Prevention of HIV-1 infection with early antiretroviral therapy. N. Engl. J. Med..

[5] Rodger A.J., Cambiano V., Bruun T., Vernazza P., Collins S., van Lunzen J., Corbelli G.M., Estrada V., Geretti A.M., Beloukas A. (2016). Sexual activity without condoms and risk of HIV transmission in serodifferent couples when the HIV-positive partner is using suppressive antiretroviral therapy. JAMA.

[6] Bavinton B.R., Pinto A.N., Phanuphak N., Grinsztejn B., Prestage G.P., Zablotska-Manos I.B., Jin F., Fairley C.K., Moore R., Roth N. (2018). Viral suppression and HIV transmission in serodiscordant male couples: An international, prospective, observational, cohort study. Lancet HIV.

[7] Squires K., Kityo C., Hodder S., Johnson M., Voronin E., Hagins D., Avihingsanon A., Koenig E., Jiang S., White K. (2016). Integrase inhibitor versus protease inhibitor based regimen for HIV-1 infected women (WAVES): A randomised, controlled, double-blind, phase 3 study. Lancet HIV.

[8] Gebrekristos H.T., Mlisana K.P., Karim Q.A. (2005). Patients’ readiness to start highly active antiretroviral treatment for HIV. BMJ.

[9] Koenig S.P., Dorvil N., Dévieux J.G., Hedt-Gauthier B.L., Riviere C., Faustin M., Lavoile K., Perodin C., Apollon A., Duverger L. (2017). Same-day HIV testing with initiation of antiretroviral therapy versus standard care for persons living with HIV: A randomized unblinded trial. PLoS Med..

[10] Girometti N., Nwokolo N., McOwan A., Whitlock G. (2017). Outcomes of acutely HIV-1-infected individuals following rapid antiretroviral therapy initiation. Antivir Ther..

[11] Labhardt N.D., Ringera I., Lejone T.I., Klimkait T., Muhairwe J., Amstutz A., Glass T.R. (2018). Effect of offering same-day ART vs usual health facility referral during home-based HIV testing on linkage to care and viral suppression among adults with HIV in Lesotho. JAMA.

[12] Rosen S., Maskew M., Larson B.A., Brennan A.T., Tsikhutsu I., Fox M.P., Vezi L., Bii M., Venter W.D.F. (2019). Simplified clinical algorithm for identifying patients eligible for same-day HIV treatment initiation (SLATE): Results from an individually randomized trial in South Africa and Kenya. PLoS Med..

[13] Cuzin L., Cotte L., Delpierre C., Allavena C., Valantin M.A., Rey D., Delobel P., Pugliese P., Raffi F., Cabié A. (2019). Too fast to stay on track? Shorter time to first anti-retroviral regimen is not associated with better retention in care in the French Dat’AIDS cohort. PLoS ONE.

[14] Davey D.J., Kehoe K., Serrao C., Prins M., Mkhize K., Hlophe K., Sejake S., Malone T. (2020). Same-day antiretroviral therapy is associated with increased loss to follow-up in South African public health facilities: A prospective cohort study of patients diagnosed with HIV. J. Int. AIDS Soc..

[15] Pilcher C.D., Ospina-Norvell C., Dasgupta A., Jones D., Hartogenesis S.T., Calderon F., Demicco E., Geng E., Gandhi M., Havlir D.V. (2017). The effect of same-day observed initiation of antiretroviral therapy on HIV viral load and treatment outcomes in a U.S. public health setting. J. Acquir. Immune Defic. Syndr..

[16] Singh S., Mitsch A., Wu B. (2017). HIV care outcomes among men who have sex with men with diagnosed HIV infection—United States, 2015. Morb. Mortal. Wkly. Rep..

[17] World Health Organization (2017). Guidelines for Managing Advanced HIV Disease and Rapid Initiation of Antiretroviral Therapy.

[18] Amanyire G., Semitala F.C., Namusobya J., Katuramu R., Kampiire L., Wallenta J., Charlebois E., Camlin C., Kahn J., Chang W. (2016). Effects of a multicomponent intervention to streamline initiation of antiretroviral therapy in Africa: A stepped-wedge cluster-randomised trial. Lancet HIV.

[19] Ford N., Migone C., Calmy A., Kerschberger B., Kanters S., Nsanzimana S., Mills E.J., Meintjes G., Vitoria M., Doherty M. (2018). Benefits and risks of rapid initiation of antiretroviral therapy. AIDS.

[20] Dang B.N., Westbrook R.A., Hartman C.M., Giordano T.P. (2016). Retaining HIV patients in care: The role of initial patient care experiences. AIDS Behav..

[21] Helova A., Akama E., Bukusi E.A., Musoke P., Nalwa W.Z., Odeny T.A., Onono M., Spangler S.A., Turan J.M., Wang I. (2017). Health facility challenges to the provision of Option B+ in western Kenya: A qualitative study. Health Policy Plan..

[22] Katirayi L., Namadingo H., Phiri M., Bobrow E.A., Ahimbisibwe A., Berhan A.Y., Buono N., Moland K.M., Tylleskär T. (2016). HIV-positive pregnant and postpartum women’s perspectives about Option B+ in Malawi: A qualitative study. J. Int. AIDS Soc..

[23] Maek-a-nantawat W., Phanuphak N., Teeratakulpisarn N., Pakam C., Kanteeranon T., Chaiya O., Mansawat T., Teeratakulpisarn S., Nonenoy S., Ananworanich J. (2014). Attitudes toward, and interest in, the test-and-treat strategy for HIV prevention among Thai men who have sex with men. AIDS Care.

[24] Mateo-Urdiales A., Johnson S., Smith R., Nachega J.B., Eshun-Wilson I. (2019). Rapid initiation of antiretroviral therapy for people living with HIV. Cochrane. Database Syst. Rev..

[25] Coffey S., Bacchetti P., Sachdev D., Bacon O., Jones D., Ospina-Norvell C., Torres S., Lynch E., Camp C., Mercer-Slomoff R. (2019). RAPID antiretroviral therapy: High virologic suppression rates with immediate antiretroviral therapy initiation in a vulnerable urban clinic population. AIDS.

[26] Bacon O., Chin J., Cohen S.E., Sachdev D., Coffey S., Scheer S., Hessol N.A., Buchbinder S., Havlir D.V., Hsu L. (2020). Decreased Time from HIV diagnosis to care, ART Initiation, and virologic Suppression during the citywide RAPID initiative in San Francisco. Clin. Infect. Dis..

[27] Hoenigl M., Chaillon A., Moore D.J., Morris S.R., Mehta S.R., Gianella S., Amico K.R., Little S.J. (2016). Rapid HIV viral load suppression in those initiating antiretroviral therapy at first visit after HIV diagnosis. Sci. Rep..

[28] Colasanti J., Sumitani J., Mehta C.C., Zhang Y., Nguyen M.L., Del Rio C., Armstrong W.S. (2018). Implementation of a rapid entry program decreases time to viral suppression among vulnerable persons living with HIV in the Southern United States. Open Forum Infect. Dis..

[29] Rodriguez A.E., Wawrzyniak A.J., Tookes H.E., Vidal M.G., Soni M., Mwanyanwu R., Goldberg D., Freeman R., Villamizar K., Alcaide M.L. (2019). Implementation of an immediate HIV treatment initiation program in a public/academic medical center in the U.S. South: The Miami test and treat rapid response program. AIDS Behav..

[30] Halperin J., Conner K., Butler I., Zeng P., Myers L., Clark R., Van Sickels N. (2019). A care continuum of immediate ART for newly diagnosed patients and patients presenting later to care at a federally qualified health center in New Orleans. Open Forum Infect. Dis..

[31] Centers for Disease Control and Prevention (CDC) (2019). Diagnoses of HIV Infection in the United States and Dependent areas, 2018 (Preliminary). HIV Surveill. Rep..

[32] Centers for Disease Control and Prevention (CDC) (2019). Estimated incidence and prevalence in the United States 2010-2016. Hiv Surveill. Suppl. Rep..

[33] Hess K.L., Hu X., Lansky A., Mermin J., Hall H.I. (2017). Lifetime risk of a diagnosis of HIV infection in the United States. Ann. Epidemiol..

[34] Clark H., Babu A.S., Wiewel E.W., Opoku J., Crepaz N. (2017). Diagnosed HIV infection in transgender adults and adolescents: Results from the National HIV Surveillance System, 2009-2014. AIDS Behav..

[35] Department of Health and Human Services Panel on Antiretroviral Guidelines for Adults and Adolescents. Guidelines for the Use of Antiretroviral Agents in Adults and Adolescents with HIV. http://www.aidsinfo.nih.gov/ContentFiles/AdultandAdolescentGL.pdf.

[36] Braveman P., Gruskin S. (2003). Defining equity in health. J. Epidemiol. Community Health.

[37] Turan B., Hatcher A.M., Weiser S.D., Johnson M.O., Rice W.S., Turan J.M. (2017). Framing mechanisms linking HIV-related stigma, adherence to treatment, and health outcomes. Am. J. Public Health.

[38] Quinn K., Voisin D.R., Bouris A., Jaffe K. (2017). Multiple dimensions of stigma and health related factors among young black men who have sex with men. AIDS Behav..

[39] Kutnick A.H., Gwadz M.V., Cleland C.M., Leonard N.R., Freeman R., Ritchie A.S., McCright-Gill T., Ha K., Martinez B.Y., the BCAP Collaborative Research Team (2017). It’s a process: Reactions to HIV diagnosis and engagement in HIV care among high-risk heterosexuals. Front. Public Health.

[40] Rueda S., Mitra S., Chen S., Gogolishvili D., Globerman J., Chambers L., Wilson M., Logie C.H., Shi Q., Morassaei S. (2016). Examining the associations between HIV-related stigma and health outcomes in people living with HIV/AIDS: A series of meta-analyses. BMJ Open.

[41] Katz I.T., Ryu A.E., Onuegbu A.G., Psaros C., Weiser S.D., Bangsberg D.R., Tsai A.C. (2013). Impact of HIV-related stigma on treatment adherence: Systematic review and meta-synthesis. J. Int. AIDS Soc..

[42] Washington H.A. (2006). Medical Apartheid: The Dark History of Medical Experimentation on Black Americans from Colonial Times to the Present.

[43] Gaston G.B., Alleyne-Green B. (2013). The impact of African Americans’ beliefs about HIV medical care on treatment adherence: A systematic review and recommendations for interventions. AIDS Behav..

[44] Kalichman S.C., Eaton L., Kalichman M.O., Grebler T., Merely C., Welles B. (2016). Race-based medical mistrust, medication beliefs and HIV treatment adherence: Test of a mediation model in people living with HIV/AIDS. J. Behav. Med..

[45] Eaton L.A., Driffin D.D., Kegler C., Smith H., Conway-Washington C., White D., Cherry C. (2015). The role of stigma and medical mistrust in the routine health care engagement of black men who have sex with men. Am. J. Public Health.

[46] Grimes R.M., Grimes D.E. (2010). Readiness: The state of the science (or the lack thereof). Curr. HIV/AIDS Rep..

[47] Freeman R., Gwadz M.V., Silverman E., Kutnick A., Leonard N.R., Ritchie A.S., Reed J., Martinez B.Y. (2017). Critical race theory as a tool for understanding poor engagement along the HIV care continuum among African American/Black and Hispanic persons living with HIV in the United States: A qualitative exploration. Int. J. Equity Health.

[48] Boyd M.A., Boffito M., Castagna A., Estrada V. (2019). Rapid initiation of antiretroviral therapy at HIV diagnosis: Definition, process, knowledge gaps. HIV Med..

[49] Hatzenbuehler M.L. (2016). Structural stigma: Research evidence and implications for psychological science. Am. Psychol..

[50] Rebeiro P.F., McPherson T.D., Goggins K.M., Turner M., Bebway S.S., Rogers W.B., Brinkley-Rubinstein L., Person A.K., Sterling T.R., Kripalani S. (2018). Health literacy and demographic disparities in HIV care continuum outcomes. AIDS Behav..

[51] Pence B.W., Mugavero M.J., Carter T.J., Leserman J., Thielman N.M., Raper J.L., Proeschold-Bell R.J., Reif S., Whetten K. (2012). Childhood trauma and health outcomes in HIV-infected patients: An exploration of causal pathways. J. Acquir. Immune Defic. Syndr..

[52] Nightingale V.R., Sher T.G., Mattson M., Thilges S., Hansen N.B. (2011). The effects of traumatic stressors and HIV-related trauma symptoms on health and health related quality of life. AIDS Behav..

[53] Centers for Disease Control and Prevention (CDC) Factsheet. https://www.cdc.gov/masstrauma/factsheets/public/coping.pdf.

[54] Yelverton V., Ostermann J., Hobbie A., Madut D., Thielman N. (2018). A mixed methods approach to understanding antiretroviral treatment preferences: What do patients really want?. AIDS Patient Care STDS.

[55] Nyblade L., Stockton M.A., Giger K., Bond V., Ekstrand M.L., Mc Lean R., Mitchell E.M., Nelson L.E., Sapag J.C., Siraprapasiri T. (2019). Stigma in health facilities: Why it matters and how we can change it. BMC Med..

[56] Ware N.C. (2019). Qualitative contributions to implementation research on HIV prevention and treatment. J Acquir Immune Defic Syndr..

